# Gluten Detection Methods and Their Critical Role in Assuring Safe Diets for Celiac Patients

**DOI:** 10.3390/nu11122920

**Published:** 2019-12-02

**Authors:** Claudia E. Osorio, Jaime H. Mejías, Sachin Rustgi

**Affiliations:** 1 Agriaquaculture Nutritional Genomic Center, CGNA, Las Heras 350, Temuco 4781158, Chile; 2 Centro Regional de Investigación Carillanca, Instituto de Investigaciones Agropecuarias INIA, Temuco 4880000, Chile; 3 Department of Crop and Soil Sciences, Washington State University, Pullman, WA 99164, USA; 4 Department of Plant and Environmental Sciences, School of Health Research, Clemson University Pee Dee Research and Education Center, Florence, SC 29506, USA

**Keywords:** gluten contamination, celiac disease, gluten detection, food labeling, aptamers, prolamins, wheat

## Abstract

Celiac disease, wheat sensitivity, and allergy represent three different reactions, which may occur in genetically predisposed individuals on the ingestion of wheat and derived products with various manifestations. Improvements in the disease diagnostics and understanding of disease etiology unveiled that these disorders are widespread around the globe affecting about 7% of the population. The only known treatment so far is a life-long gluten-free diet, which is almost impossible to follow because of the contamination of allegedly “gluten-free” products. Accidental contamination of inherently gluten-free products could take place at any level from field to shelf because of the ubiquity of these proteins/grains. Gluten contamination of allegedly “gluten-free” products is a constant threat to celiac patients and a major health concern. Several detection procedures have been proposed to determine the level of contamination in products for celiac patients. The present article aims to review the advantages and disadvantages of different gluten detection methods, with emphasis on the recent technology that allows identification of the immunogenic-gluten peptides without the use of antibodies. The possibility to detect gluten contamination by different approaches with similar or better detection efficiency in different raw and processed foods will guarantee the safety of the foods for celiac patients.

## 1. Introduction

Wheat feeds one-quarter of the annual worldwide demand for plant proteins (60 metric tons) and has been the source of nutrition since the dawn of human civilization [1,2,3,4]. It is not only a primary staple worldwide. But it is also responsible for numerous foodborne disorders, which remained one of the major causes of premature deaths in the most resource-deprived parts of the world since the prehistoric-times [3,5,6,7,8]. Given its vast influence on human health, domestication of bread wheat and its subsequent industrialization has been considered a “mistake of evolution” that created conditions for human diseases related to gluten exposure [7,9,10]. The word “gluten” refers to a complex mixture of proline and glutamine-rich seed-storage proteins that serves as fuel for multiple disorders [11,12,13,14,15,16]. Different overlapping or non-overlapping epitopes have been shown to elicit various reactions in different individuals in accordance to their genetic constitutions [14,17,18,19,20]. Gluten-intake in sensitive individuals can lead to gastrointestinal, neurological, and fatal symptoms such as non-Hodgkin lymphoma [21,22,23]. These symptoms can be classified grossly into celiac disease, wheat sensitivity, and allergy [11,23,24], with the celiac disease being the most prevalent gastrointestinal disorder [23]. In celiac disease, the response to gluten is mediated by the adaptive immune system and the induction of autoantibodies against the indigestible gluten peptides and tissue transglutaminase 2 (tTG2), an enzyme involved in tissue homeostasis [25]. The tTG2 is also responsible for chemical modification of gluten peptides, which enhance their recognition by the immune system. In this process, the faulty immune system in genetically predisposed individuals recognizes tTG2 as an enemy and triggers an autoimmune response against it [14,26]. Because of their mode of action, gluten peptides were compared with the non-replicating pathogen [27]. Since like pathogens, these peptides evade “host” defenses by escaping digestion through gastrointestinal enzymes, invade the intestinal epithelium, take a more aggressive form after the modification by tTG2, and trigger a cascade of reactions leading to the intestinal and extra-intestinal symptoms [14,27,28]. The first reaction initiated by gluten-peptides gets amplified to take a more severe form of an autoimmune disorder upon recognition of tTG2 by the immune system as antigen [29,30]. The second kind of reaction is gluten-allergy, which involves both the innate and adaptive immune systems [23,31]. It is a quick reaction against the external allergen within few minutes to hours after ingestion or inhalation and results in a variety of symptoms such as dermatitis, anaphylaxis, etc., [32]. The third kind of reaction known as wheat sensitivity involves the innate immune system and is associated with diverse symptoms ranging from fatigue, distress, depression, and migraines to gastrointestinal disorders [31,33].

The only known treatment for gluten-associated disorders is a life-long wheat exclusion diet [14,16]. Such a diet is difficult to follow because of the unintended contamination of “gluten-free” products, improper labeling, social constraints, and ubiquity of gluten proteins in raw or cooked food and pharmaceuticals [34,35,36]. Thus, accidental gluten encounters are likely [37,38]. Different celiac patients show sensitivity to different gluten proteins [9,39]. Besides, different individuals show different tolerance levels for gluten intake. In general, celiac patients were shown to tolerate up to 20 mg gluten in a kg of food consumed in a day [40,41]. Therefore, it is crucial to precisely monitor the gluten content of the food prepared for celiac patients and to maintain gluten-levels below the prescribed limits in their diets [42].

According to the Codex definition, any food product containing >20 mg/kg gluten cannot be considered or labeled as “gluten-free” [43]. Because of the gluten contamination, many inherently gluten-free products (derived from corn, rice, millet, oats, etc.,) cannot be consumed by celiac patients [37,38]. These products, if misbranded as “gluten-free” and used by the celiac patients, will result in recurrence of symptoms [34]. The gluten contamination can take place at any level from field to the shelf during harvesting, transportation, and/or processing [44,45,46]. In bakeries where gluten is present ubiquitously, it is almost impossible to decontaminate all equipment, thus unconsciously contaminate the “gluten-free” products [47]. For instance, in analysis of R5 antibody-based enzyme-linked immunosorbent assay (ELISA) of 22 commonly available, “gluten-free” commodities including grains, seeds, and flours, seven showed mean gluten levels of more than 20 mg/kg [44]. Antibody-based methods of gluten detection, specifically those relying on R5 and G12 antibodies, are also endorsed by the Prolamin Working Group of the Codex Alimentarius Commission to test gluten contamination in raw and processed food samples (cf. Table 1) [48]. There is sufficient evidence to support that even products derived from inherently gluten-free grains cannot be considered safe under the proposed FDA rules for gluten-free labeling [43,44,49,50,51,52,53,54,55]. As most of these surveys were performed on the raw material, it is very likely, that processed food or convenience products, which have more chances of getting contaminated, will show even higher gluten contamination levels. Besides, most of the existing detection methods employ the sandwich ELISA system, which suffers from the following inherent problems. i) Sandwich ELISA can only be applied to antigens larger than 5-kDa in size and with at least two sterically distant epitopes for their binding and detection by capture and detection antibodies. It thus is unsuitable for the detection of hydrolyzed protein products. ii) The detection limit for most of the assays is 10 mg/kg, with a high error rate close to the lower detection limits. iii) Biased detection of one family of proteins over others, leading to the overestimation of one and underestimation of the other protein family. iv) Protein contaminations in heat-processed food samples (especially glutenins) are difficult to detect in these assays. Given the problem associated with the commonly used gluten detection method, the major objective of this review is to look into other gluten detection methods, with better sensitivity and possibility for implementation in the food industry.

## 2. Celiac Disease Prevalence

The availability of better tests such as serological analysis for antibodies against tissue transglutaminase (tTG), deamidated gliadin peptide (DGP), and anti-endomysial antibodies (EMA), as well as small bowel biopsy, have improved celiac disease (CD) diagnosis and distinction between CD and non-celiac wheat sensitivity (NCWS) [26,56,57,58]. Worldwide prevalence of celiac disease has been documented to range between 0.5 to 1.7% [59,60]. It is noteworthy that the prevalence of CD has increased over the decades, with an incidence rate of 0.6% in the 1990s to 0.8% between 2001 to 2016. Better diagnostics and health awareness might be responsible for this increased CD prevalence. In the northern hemisphere, the incidence in adults diagnosed by biopsy ranges from 0.96% in Canada, 1% in the United States to ~2% in Europe [61,62]. In the southern hemisphere, the once thought to be the “celiac-free” region of the world has reported having similar CD prevalence to European countries. The latest surveys have established an equal incidence of celiac disease in Asia and Africa [59,63,64]. Specifically, in India, a celiac disease frequency of 1.04% was reported [65,66,67]. In some provinces of China, the CD prevalence, similar to the global incidence of two individuals diagnosed every hundred, was reported [68,69]. In Northern Africa, the CD prevalence of about 0.3 to 5.6% was observed. In Australia and New Zealand, a CD incidence of 0.5% and 1.2 %, respectively, was reported. In South America, specifically Argentina and Brazil, a range of 0.2 to 0.6% was observed. Whereas, in Mexico, a higher rate close to 3% of CD occurrence was reported [59]. It is possible, over the years, with the advancement in technology and better diagnostics, several patients currently considered to suffer from NCWS will be diagnosed with celiac disease, leading to the changes in our understanding of CD prevalence. The prevalence of NCWS has been documented to range between 0.6 to 13% in the general population. However, because of the lack of diagnostic criteria, biomarkers, and misconceptions on self-diagnosis, the actual prevalence of NCWS cannot be established with a level of confidence today [70]. These epidemiological studies have suggested that gluten-associated disorders are common around the globe, and reflected toward a need for the “gluten-free” products. However, given the market trend and consumer safety in mind, it is imperative to ascertain that the products labeled as “gluten-free” should have and maintain a gluten-free status.

## 3. Hidden Gluten or Gluten Contamination

The products containing hidden gluten include sausages, fish fingers, cheese spreads, soups, sauces, mixed seasonings, mincemeats, some medications, and food supplements like vitamin preparations [34]. Some beverages like real ales, beers, and stout are also contaminated with gluten generally [36]. The primary causes of contamination are either use of common machinery during the harvest, transportation, and processing or the use of shared storage space [71]. Mixed, parallel or sequential cultivation of gluten-containing and gluten-free cereals could also lead to contamination of the inherently gluten-free grains [49]. Contamination is unavoidable if the same milling equipment is used for gluten-free and gluten-containing grains. Interestingly there is no legislature in place regarding the maximum levels of foreign grains in gluten-free cereals. However, in general, 2% of other grains are used as the maximum limit. 

The ubiquitous nature of gluten constitutes a global problem. Contamination of food has been reported as early as 1987 in Sweden, United Kingdom, Canada, South Africa, Brazil, the United States, Italy, Australia, and Ireland [46]. The existence of gluten contamination was reported in industrial and non-industrial products, with or without a “gluten-free” label, with an overall prevalence of 23.2% [46]. Besides the methodological limitations, such as different detection methods, the experimental design, or non-consensus on the gluten reference material, there is an agreement on the importance of strict regulations to check gluten contamination. Studies conducted over time have shown a decrease in the number of gluten-contaminated samples worldwide [72,73]. After analysis of a fifteen-year period, more significant decrease was observed in cereals and additives, which were down from 37.7% and 33.0% of gluten contaminated samples to 7% and 2.6%, respectively [72,74]. In the European Union, 55% of the gluten-free food products were found contaminated in the period between 2003–2005. In contrast, only 19% of the food products were found contaminated in the period between 2013 to 2016, which is concordant with the codex revision implementation followed by Central and Western European countries [72]. This revision was critical for snacks and yeasts, where gluten level was found unsafe for celiac patients. It forced the yeast bakery product manufacturers to change their practices to ensure gluten control in their products. During recent years, the development of non-invasive methods to detect gluten-related disorders [75], allowed patients to check the quality of the products and demanding better and reliable food labeling.

## 4. Gluten Threshold or Tolerance Level

A gluten-free diet does not necessarily mean “zero gluten” because low levels of gluten seem to be tolerated by the patients. In consideration of the worldwide prevalence of the celiac disease, establishing a threshold for gluten intake by the patients is a matter of general interest. Albeit several studies were conducted in the past to determine the effect of low gluten intake in patients with celiac disease, a more detailed study is required to reach a consensus. In the 1980s, by studying the toxicity and time response to the gliadin doses in a single patient, the conclusion was reached that in between 10 to 100 mg of gliadins induce slight to no changes in the small intestine morphology [76]. Whereas, 500 mg to 1 g gliadin doses respectively cause moderate to extensive damage to the small intestine. Later on, in a more detailed study, the same group of researchers reported that 2.4–4.8 mg gluten per day caused no damage to the jejunal morphometry over an observation period of 1–6 weeks [77]. In an earlier study, Ejderhamn and collaborators reached a similar conclusion that a daily intake of 4–14 mg gliadin does not cause morphological changes in the small bowel mucosa of celiac patients on an abstinent diet [78]. Two Finnish groups also made similar observations, however, with slightly higher daily doses of gluten (20–36 mg) in the celiac patients on the gluten-free diets [79,80]. In a later study, even a higher amount (10 to 100 mg) of daily gluten intake was reported to be safe for celiac patient consumption [81]. On the other hand, Catassi et al. [82] demonstrated that 100 mg of the gliadins per day cause deterioration of the small intestine architecture, and the effects are more pronounced with 500 mg gliadin per day. Despite several studies conducted in the past, a consensus on the critical limit or threshold for gluten intake has not been reached. This large variability in response to gluten among celiac patients was also witnessed in a double-blind placebo-controlled multicenter investigation for the gluten toxicity (10–50 mg/day) on 40 celiac patients. In this study, the patients were administered daily with a capsule containing 0 mg, 10 mg, or 50 mg of gluten for 90 days and analyzed for clinical, serological, and histological changes in their small intestines. In this study, the authors reported a large variability among patients in terms of gluten sensitivity. Some patients showed intestinal symptoms already after ingesting daily 10 mg of gluten while other patients showed no histological symptoms even after three months on 50 mg of the gluten daily. Additionally, in a similar study, it was reported that 50 mg of daily gluten intake for three months is sufficient to cause significant damage to the intestinal morphology of the tested celiac patients [83].

Because of the observed variability, different countries adapted different labeling regimens for the products sold for celiac patients. In Europe, two labeling categories have been defined as (i) “Gluten-free” and (ii) “Very low gluten.” To be listed under the first category, the products must contain less than 20 mg/kg gluten, and under the second category, it should contain less than 100 mg/kg gluten. Similarly, FSANZ (the Food Standards of Australia and New Zealand) recognizes two classes of foods, “gluten-free foods” with no detectable gluten and “low-gluten foods” with no more than 200 mg/kg gluten. Canada followed a more straightforward approach where a “gluten-free” label means food without wheat, oats, barley, rye, triticale, and their parts. More recently, the United States Food and Drug Administration also set a limit of 20 mg/kg gluten for the “gluten-free” products” [84].

The decision on the threshold depends mainly on two factors (i) the minimum toxic dose, and (ii) the amount of “gluten-free” product(s) consumed. The results of the food challenge studies indicated that 200 mg/kg is not a safe threshold as the gluten intake limit of 50 mg/kg could be reached with the consumption of 250 g of allegedly gluten-free product(s). A 100-mg/kg limit that allows 10 mg gluten in 100 g of food is also impractical, as in Europe, consumption of gluten-free products could be as high as 500 g per day [85]. However, the threshold of 20 mg/kg keeps the intake of gluten from “gluten-free” food (processed/unprocessed), well below the 50 mg amount. Thus, it allows a safety margin for the variable gluten sensitivities and dietary habits of the different patients. 

## 5. Food Labeling

Gluten-sensitive individuals rely mostly on product labels to make diet decisions. Therefore, it is imperative to label all food ingredients, particularly in the case of the composite pre-packed foods. The food ingredients that cause intolerance and/or allergy are documented in the “list of hypersensitivity” complied by the Codex Commission. This list includes the gluten-containing cereals, Crustacea, eggs, fish, peanuts, milk, tree nuts, and derivatives of the items listed above [86]. Gluten is not a permitted food additive in the European countries; therefore, the label must include all ingredients [87]. Ingredients such as soluble wheat proteins and starches are, however, used without any declaration in several food products. Therefore, the Codex Commission declared that gluten-ingredients from wheat, including Spelt (*Triticum spelta* L.), Khorasan, or Kamut (*T. polonicum* L.), durum, einkorn (*T. monococcum*), barley, rye, triticale, tritordium, and their hybrids should be declared [84]. The primary concern at present is the misbranding of single or multi-ingredient food products as gluten-free without proper testing, specifically in cases where the product is derived from the inherently gluten-free grains [88]. Thus, to brand these products gluten-free, it is crucial to test and ascertain that the gluten level stays below the prescribed limit of 20 mg/kg in them.

## 6. Available Detection Methods

Over the years, several gluten-detection and quantification methods have been developed and tested using the gluten-containing and/or spiked samples. These procedures can be grossly classified into genomic, proteomic, and immunological methods [89]. The pros and cons of using these methods are discussed in this section. 

Among genomic methods, PCR (polymerase chain reaction)-based assay relies on the determination of specific DNA sequences. These methods are more sensitive by several orders of magnitude than the protein-assays. The PCR-based assay was first applied by Allmann et al. [90] to test 35 different food samples, including bakery additives and heated as well as processed food samples. In this study, wheat starch having low gliadin content was found positive by PCR, albeit the pure gliadins or glutenins, used as a food additive, could not be detected. In a separate study, oat samples spiked with wheat gluten were tested simultaneously with PCR and R5 ELISA, and PCR showed ten times more sensitivity than R5 ELISA [91]. Later, Dahinden et al. [92] developed a quantitative competitive (QC-) PCR system to detect wheat, rye, and barley contaminations. The QC-PCR was applied to 15 gluten-free commodities, which gave results comparable to the ELISA test performed using the R5 monoclonal antibody (mAb). Similar conclusions were reached in another study performed using real-time PCR [93]. In the same year, Henterich et al. [94] developed a real-time immuno-PCR assay for gliadin detection, where an R5 mAb was conjugated with an oligonucleotide. The results showed 30-fold more sensitivity over ELISA. In a later experiment, Mujico and collaborators [95] developed a highly sensitive RT-PCR based system for gluten detection in raw and processed samples, which exhibited more sensitivity than R5 ELISA. A comparison of the results obtained over a six-year period between laboratories using PCR and ELISA for wheat gluten detection showed that PCR gave no false positives. In contract, ELISA detected 2% false positives, specifically in processed food samples [96]. Despite the high sensitivity, PCR assays cannot be applied to the hydrolyzed products such as beer, syrup, and malt extracts for the determination of their gluten content. 

The relatively more direct and precise method for gluten detection and quantification is matrix-assisted laser desorption/ionization time-of-flight mass spectrometry (MALDI-TOF MS). It can simultaneously measure the protein and protein hydrolysate ranging in size from 1000 to 100,000 Daltons without a need for chromatographic purification [97]. Additionally, this technique allows reliable determination of protein levels as low as 0.01 mg/ml in the food samples [97]. This method was first applied to test 30 “gluten-free” food samples, and the results were comparable with that of ELISA [98], with an added advantage, as it allowed determination of the contamination source [99]. MALDI-TOF MS is a highly sensitive non-immunological approach for the detection and quantification of gluten contamination in food samples. However, its application requires highly expensive specialized equipment, and the method is applicable only to make semi-quantitative measurements [100]. Coupling HPLC could overcome this limitation to tandem mass spectrometry (LC-MS/MS) [89]. However, there are a few points of consideration, such as the type of peptidase treatment needed for gluten detection, the reference material for gluten quantification, and the extraction of proteins from the food matrix. Earlier protocols allowed identification of gluten source in beer made from wheat, barley, or buckwheat [101]. And, the following methods allowed gluten quantitation in the range of 0.01 to 100 mg/kg in raw and processed food samples [102]. Using LC-MS/MS proteomic profiles were obtained for 60 beer samples [103], where each hordein fraction was expressed as percentages of prolamin content [104]. Also, in beer samples, differences between ELISA and mass spectrometry were observed, where samples detected negative for hordeins in ELISA were found positive for B-hordein fragments in MS analysis [105]. Similarly, targeted LC-MS/MS analysis allowed detection of wheat gluten peptides at concentrations of 10 and 15 mg/kg, respectively, in chymotrypsin and trypsin treated oat and soy flour samples [106,107]. 

Column chromatography is another method that has been used extensively for characterization, separation, and quantification of the cereal seed-storage proteins. Gel permeation (GP) chromatography, which separates proteins based on their molecular weights, and reverse-phase (RP) chromatography that separates proteins according to their hydrophobicities, are the most commonly used methods [108]. These procedures have advantages in terms of speed (often 30 min runs) and detection capability, which is as low as 1-2 mg gluten. These methods have been used successfully in determining gluten contamination in 23 starch samples with gliadin content of 15 to 574 mg/kg [109]. Although this method can be used to access gluten contamination reliably, it has the disadvantage of being unable to differentiate between gluten and non-gluten proteins in the complex food samples. 

More recently, the applicability of near-infrared (NIR) spectroscopy for the detection of gluten contamination in gluten-free products was proposed [110]. For gluten detection and quantification, NIR spectroscopy was combined with chemometric techniques. However useful, this technique relies on the development of a suitable calibration model, which depends on the availability of a large number of samples characterized by other methods to carry gluten contamination to serve as a training set. Therefore, the availability of a training set is a prerequisite to calibrate the NIR equipment to test unknown samples for gluten contamination, which may or may not be available to the user.

The more versatile and commonly accepted assays are immunological assays, in particular ELISA. Owing to the sensitivity and speed of detection, the Codex Committee on Methods of Analysis and Sampling has endorsed these methods [111]. Several variations of these methods have been developed over the years. Several antibodies (monoclonal and polyclonal) and a variety of commercial kits are available in the market to perform these assays [89]. The commonly used ELISA systems can be grossly divided into two categories: the sandwich ELISA and the competitive ELISA [89]. In the sandwich ELISA the antigen is sandwiched between two antibodies, one immobilized to the walls of the microtiter plate (capture antibody) and the other coupled with an enzyme (detection antibody). The sandwich ELISA is only suitable for large antigens because the antigen should have at least two separate epitopes to bind both antibodies. Thus, this ELISA system is not an appropriate choice for partially hydrolyzed gluten samples like in the sourdough products, malt, and beer. The other ELISA system is competitive ELISA, which is suitable for the detection of small-sized antigens with a single epitope. In this system, labeled and unlabeled antigen is applied to immobilized antibody, where they compete for the antibody binding sites. After washing out the unbound antigen, the quantity of the labeled antigen is determined by adding the enzyme-substrate and measuring the intensity of the colored end product, which corresponds with the quantity of the labeled antigen. The major problem associated with both of the ELISA systems is the determination of gluten contamination in heat-processed food samples, which cause conformational changes to the antigen masking or modifying the antibody recognition site(s) [89]. It has been documented that the α/β- and γ-gliadins lose 49 to 67% of the original reactivity after the heat treatment, while the ω-gliadins remain largely unaffected, i.e., they only lose 7% of reactivity [112,113]. The commercially available prolamin detection kits are summarized in Table 1. 

There are, however, several shortcomings associated with the use of ELISA, the most important being the appearance of false positives due to the requirement of an enzymatic reaction for antigen detection/quantification and non-specific binding of antigen to the plate. To overcome these challenges, during the last decade, there have been efforts to develop enzyme-free assays capable of fast quantification, and requirement of the small amount of sample for analysis. In this area, the development of nanomaterials and their use in biomolecular sensing has gained increasing importance [114]. Nanomaterials are defined as materials with a size of less than 100 nm in at least one dimension [115]. The nanoparticles possess unique properties because of their small size, composition, and high specific surface area (the total surface area per unit of mass), allowing them to exhibit extraordinary reactivity. Furthermore, they present unique optical, electrochemical, and magnetic properties, which have been extensively exploited for capture, recognition, and quantification of chemical targets and biomolecules such as proteins [114]. One of the most important properties of the nanoparticles is the possibility of modifying their surface chemistry. Conjugation of nanoparticles with different surface binding chemical groups allows the designing of nanoprobes that can be used in biomolecular sensing of specific targets with many advantages over conventional assays [116,117]. Noble metal nanoparticles have been used lately for the detection of food allergens with promising results. For instance, immunosensors were developed using modified gold nanoparticles for gliadin detection [118,119,120]. The specificity of the assays was tested against flour and breakfast cereals containing a matrix from different grains. In the case of carbon/nanogold screen-printed electrodes directly functionalized with gliadin, the detection was tested against four products, breadcrumbs, durum wheat pasta, crackers, and biscuits. In all cases, the immunosensor tests exhibited more specificity compared with R5 ELISA (RIDASCREEN), detecting lower amounts of gliadins in selected foods, on average 8,500 mg/kg less [118]. A different approach was taken by Chu and collaborators, who used a label-free gliadin immunosensor based on changes in the frequency of a quartz crystal microbalance (QCM) chip. With this system, the authors were able to detect gliadin levels as low as 8 ppb in the samples [119]. Following the international Codex Alimentarius Standard [43], and using a DNA recognition system, Yin et al. were able to classify samples into “gluten-free” and “very low gluten” foods [120]. In a screen of 48 samples, this assay successfully differentiated between wheat and seven commonly used grains. The results were highly concordant with those obtained by the Association of Official Agricultural Chemists (AOAC)-approved ELISA or strip kits [120]. In a recent study, taking advantage of the dimer of the two different size silver nanoparticles linked by gliadin IgG Biot, the authors reported getting more astringent results both in terms of the limit of detection (LOD) and the limit of quantification (LOQ) [121]. When tested on corn flour and corn starch samples, this intensity depletion immunolinked assay (IDILA) showed 1000 to 10,000 times more sensitivity than ELISA, which led to better performance in terms of both LOD and LOQ [121].

**Figure 1:**
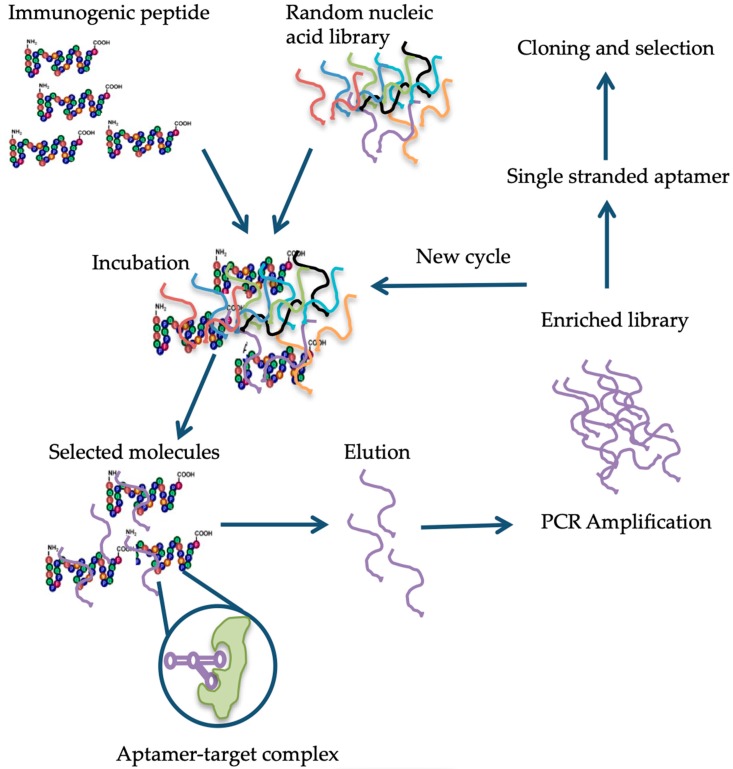
Schematic representation of the different steps in the systematic evolution of ligands by exponential enrichment (SELEX) procedure, modified from Banerjee [136] and Stoltenburg et al. [125,133].

The antibody-based detection methods suffer other drawbacks, such as these assays are not fully compatible with the extraction solutions, which lead to the denaturation of proteins [122]. In recent years, to avoid the limitations associated with antibody-based assays, aptamers were proposed as an alternative. It is generally believed that these molecules can overcome the limitations of using antibodies in the detection, identification, and quantification of specific targets due to their unique properties (cf. Table 2 and ref. [123]). The aptamers are single-stranded oligonucleotides that can bind proteins, small-molecules, and living cells with high affinity and specificity [124]. The single-stranded DNA or RNA oligonucleotide is selected in vitro via a process dubbed as the systematic evolution of ligands by exponential enrichment (SELEX) [124]. The method relies on the selection of target-specific aptamers through the repetition of the following steps: binding, partition, elution, amplification, and conditioning until the desired aptamer(s) are identified [125]. Briefly, in the case of aptamer designed for gluten detection, specifically the 33-mer immunogenic epitope, a library consisting of 10^14^-10^15^ single-stranded DNA oligonucleotides with a portion of the random nucleotide sequence is synthesized by a combinatorial chemical synthesis technique, and incubated with the target [126]. Unconjugated or low-affinity binding molecules are removed, and captured nucleic acid molecules are eluted and amplified by PCR. As a result, double-stranded PCR products are produced, which are later converted to single-stranded aptamers. The whole process is repeated several times until a group of high-affinity binding aptamers is obtained [127] (Figure 1). Aptamers are small molecules typically < 100-mers that fold into three-dimensional structures with their self-annealing properties. Target identification is due to their structure and not by their sequence (see Figure 1). Aptamer-target complexes present dissociation constants (K_d_) within the low picomolar (1 × 10^−12^ M) to nanomolar (1 × 10^−9^ M) range, which reflects toward their high binding affinity. Furthermore, target recognition is highly specific because aptamers can clearly distinguish between closely related protein targets [128].

Because of the great versatility of aptamers, these molecules have been developed against multiple targets such as cells, viruses, proteins, peptides, amino acids, and also small molecules such as metal ions, toxins, and dyes. The selectivity and high affinity of the aptamer-target interactions have been utilized for the development of biosensors and bio-detection platforms in a wide range of disciplines [127,137], which opened the possibility to their use in the detection of food contaminants. The development of specific aptamers for gluten detection, however, constitutes a challenge because of the hydrophobic nature of gluten proteins as opposed to the hydrophilic nature of nucleic acids [138]. By using magnetic beads and His-tags, an oligonucleotide able to recognize the immunogenic 33-mer peptide from gluten was developed. This aptamer performed better than ELISA and showed a LOD as low as 0.5 mg/kg [138]. A different approach was adopted by Pinto et al. [139], who immobilized wheat gliadin by adsorbing gliadins suspended in carbonate buffer onto microtiter plates, and exposing the plates to the pool of single-stranded oligonucleotides. This process has resulted in the select of an aptamer specific to gliadin 33-mer peptide, which was later used to perform a competitive apta-PCR assay for gliadin detection with a LOD value similar to ELISA [139]. However, to further improve the detection efficiency, selection of a suitable labeling system, such as 6-FAM labeling of biotinylated aptamer was tested [140]. The development of electrochemical competitive apta-sensors for gluten detection relied on the immobilization of biotinylated 33-mer peptide onto a streptavidin carbon surface, with LOD as low as 380 μg/kg [141]. When gluten content was measured in food samples, such as rolled oats or fit-snacks extracted with ethanol, with the apta-sensor, it tolerated up to 1.2% of ethanol without compromising reproducibility and performance of the assay.

When comparing methods for the detection of gluten contamination in food samples, it is vital to take into consideration the availability of appropriate reference material. In this sense, PCR-based methods have a clear advantage, as for quantification, there is no requirement to compare or calibrate against gluten reference material. In the case of chromatographic, antibody, or aptamer-based methods, one major concern in different countries that hinder the homologation of gluten food contamination in “gluten-free” food products, is the absence of a reference material designed for the specific wheat products, which will serve as the basis for analysis [142,143]. It is also true for oat, barley, and rye products [144]. Besides this concern, the use of antibody-based methods for in situ detection of gluten contamination in the food industry remains more accepted because of their user-friendliness and safety. But, there is space to incorporate other immerging technologies, such as aptamers, which can be designed to specifically bind different proteins or protein combinations with more affinity and specificity to guaranty the ease of detection and safety of food products.

## 7. Conclusions

Qualitative detection of gluten proteins has been conducted by several analytical techniques such as gel and capillary electrophoreses, PCR, QC-PCR, RP-HPLC, LC-MS, and MALDI-TOF-MS. However, these techniques, particularly those based on mass spectrometry, are relatively expensive and require specialized skills. On the other hand, quantitative detection of gluten protein in food is currently performed by immunochemical methodologies. The only commercially available method approved by the FDA and the Codex Alimentarius for gluten detection in food products are immunological tests such as ELISA. Although these tests are sensitive, they fail to detect partially hydrolyzed gluten in samples. Furthermore, there is evidence that in ELISA tests, the antibody loses its reactivity when the kit is used for gluten detection in heat-processed food samples. It is also known that reliable detection of the trace quantities of 55-67 different gluten proteins is not possible with any available single assays. A step forward is represented by aptamers, which consist of short chains of oligonucleotides that specifically recognize and bind to targets by adopting unique three-dimensional structures. Aptamers offer advancement over the antibodies. For instance, the chemical synthesis of aptamers offers extreme accuracy and reproducibility, little to no batch-to-batch variation, and allows chemical modifications to increase the substrate binding affinity further. Unlike antibodies, aptamers upon denaturation can reassume their structure within minutes, and since they are not the product of the immunological reaction, the stability under long-term storage at ambient temperature is guaranteed. In practice, aptamers function like antibodies in recognition of targets but need to be conjugated to additional components to facilitate detection. Conjugation of aptamers with functionalized nanoparticles could be achieved by cross-reacting streptavidin-coated iron oxide nanoparticles to biotin-labeled aptamers. Because of their high binding affinity, simple synthesis, performance under a range of environmental conditions, and stability in storage, aptamers are promising candidates to be used in combination with nanoparticles for gluten detection and quantification in unintentionally contaminated gluten-free products.

## Figures and Tables

**Table 1:** A list of commonly available gluten detection kits, associated antibodies, target proteins, detection procedures, and extraction systems.

Company	Neogen Corp.	R-Biopharm AG	R-Biopharm AG	Inmunología y Genética Aplicada SA	Romer Labs	Tepnel Biosystem	Morinaga Inc.
Product	Veratox	RIDA- SCREEN	Ridascreen® Gliadin Competitive	INgezim Gluten	AgraQuant® Gluten G12	Gluten assay	Wheat protein
Antibody	2 mAb	R5 mAb	R5 mAb	R5 mAb	G12 mAb	Skerritt mAb	Wheat pAb
ELISA type	Sandwich	Sandwich	Competitive	Sandwich	Sandwich	Sandwich	Sandwich
Time	30 min	1.5 h	40 min	60 min	60 min	30 min	2.5 h
Target	gliadin	ω, α/β- & γ-gliadins and LMWg	ω, α/β- & γ-gliadins and LMWg	ω, α/β- & γ-gliadins and LMWg	α gliadins	ω gliadins and HMWg	Wheat proteins
Antigen							
LOD (mg/kg)	n/a	3	1.36	3	2	1	0.3
LOQ (mg/kg)	10	5	5	10	4	10	3.12

**Table 2:** Comparison of aptamers and antibodies based on properties.

Properties	Aptamers	Antibodies	Reference
Affinity	Very high target affinity, dissociation constants from micro to picomolar range.	Lower target affinity except for some monoclonal antibodies.	[129]
Immunogenic effect	Independent of immunogenic effect, due to their in vitro production.	Immune response can fail when the target molecule, has a structure similar to an endogenous protein.	[130]
Specificity	High binding specificity, e.g., the Anti-theophyllin aptamer displayed 10,000-fold discrimination against caffeine (Theophyllin differs from caffeine by a single methyl group).	Depends on target type.	[131]
Production	In vitro.	In vivo. Use of animals or cell lines.	[132]
Consistency	Chemical synthesis, extreme accuracy, and reproducibility. Little or no batch-to-batch variation.	May have in vivo variations. Restricted to environmental conditions.	[130]
Properties	Can be optimized on demand for increasing binding affinity and specificity.	Properties cannot be changed on demand.	[126,133]
Stability	Undergo denaturation, but reversible within minutes.	Irreversible denaturation. Stable under physiological conditions	[130]
Range of targets	Combinatorial library can be produced against any type of target, even toxic targets.	Restricted to molecules that produce immunogenic effect.	[134]
Shelf-life	Stable to long-term storage at ambient temperature.	Limited shelf-life.	[132]
Functionalization	Labeling does not affect affinity.	Attachment of molecules can cause loss in affinity.	[126,133,135]
